# Intramuscular Temperature Changes in the Quadriceps Femoris Muscle After Post-Exercise Cold-Water Immersion (10°C for 10 min): A Systematic Review With Meta-Analysis

**DOI:** 10.3389/fspor.2021.660092

**Published:** 2021-05-06

**Authors:** Livia Freitag, Ron Clijsen, Carlina Deflorin, Wolfgang Taube, Jan Taeymans, Erich Hohenauer

**Affiliations:** ^1^Rehabilitation Research Laboratory 2rLab, Rehabilitation and Exercise Science Group, Department of Business Economics, Health and Social Care, University of Applied Sciences and Arts of Southern Switzerland, Landquart, Switzerland; ^2^International University of Applied Sciences THIM, Landquart, Switzerland; ^3^Department of Health, Bern University of Applied Sciences, Berne, Switzerland; ^4^Department of Movement and Sport Sciences, Vrije Universiteit Brussel, Brussels, Belgium; ^5^Department of Neurosciences and Movement Sciences, University of Fribourg, Fribourg, Switzerland; ^6^School of Sport, Health and Exercise Science, University of Portsmouth, Portsmouth, United Kingdom

**Keywords:** cold-water immersion, adipose tissue, intramuscular temperature, exercise, metabolism

## Abstract

Post-exercise cold-water immersion (CWI) is a widely accepted recovery strategy for maintaining physical performance output. However, existing review articles about the effects of CWI commonly pool data from very heterogenous study designs and thus, do rarely differentiate between different muscles, different CWI-protocols (duration, temperature, etc.), different forms of activating the muscles before CWI, and different thickness of the subcutaneous adipose tissue. This systematic review therefore aimed to investigate the effects of one particular post-exercise CWI protocol (10°C for 10 min) on intramuscular temperature changes in the quadriceps femoris muscle while accounting for skinfold thickness. An electronic search was conducted on PubMed, LIVIVO, Cochrane Library, and PEDro databases. Pooled data on intramuscular temperature changes were plotted with respect to intramuscular depth to visualize the influence of skinfold thickness. Spearman's rho (r_s_) was used to assess a possible linear association between skinfold thickness and intramuscular temperature changes. A meta-analysis was performed to investigate the effect of CWI on pre-post intramuscular temperature for each measurement depth. A total of six articles met the inclusion criteria. Maximum intramuscular temperature reduction was 6.40°C with skinfold thickness of 6.50 mm at a depth of 1 cm, 4.50°C with skinfold thickness of 11.00 mm at a depth of 2 cm, and only 1.61°C with skinfold thickness of 10.79 mm at a depth of 3 cm. However, no significant correlations between skinfold thickness and intramuscular temperature reductions were observed at a depth of 1 cm (*r*_s_ = 0.0), at 2 cm (*r*_s_ = −0.8) and at 3 cm (*r*_s_ = −0.5; all *p* > 0.05). The CWI protocol resulted in significant temperature reductions in the muscle tissue layers at 1 cm (*d* = −1.92 [95% CI: −3.01 to −0.83] and 2 cm (*d* = −1.63 [95% CI: −2.20 to −1.06]) but not at 3 cm (*p* < 0.05). Skinfold thickness and thus, subcutaneous adipose tissue, seems to influence temperature reductions in the muscle tissue only to a small degree. These findings might be useful for practitioners as they demonstrate different intramuscular temperature reductions after a specific post-exercise CWI protocol (10°C for 10 min) in the quadriceps femoris muscle.

## Introduction

Cold-water immersion (CWI) is one of the most common modalities for athletic muscle recovery (Bleakley et al., 2012). Post-exercise CWI is reported to exert a positive effect on neuromuscular performance and subjective recovery (Higgins et al., 2017). Exercising and assessment of leg muscles are commonly conducted in the area of post-exercise cooling studies, which clearly demonstrate a high relevance of optimal recovery strategies, especially for the knee extensor muscles (Bleakley et al., 2012; Costello et al., 2015). High-intensity or unaccustomed exercise can induce delayed onset of muscle soreness (DOMS), which has been investigated by several research groups over the last decade (Adamczyk et al., 2016; Fonseca et al., 2016; Hohenauer et al., 2018; Siqueira et al., 2018; de Freitas et al., 2019). CWI is reported to attenuate DOMS to a significant extent, reducing the symptoms of DOMS (up to 96 h) compared to passive control interventions (Hohenauer et al., 2015). In addition to subjective recovery variables, objective outcomes also indicate positive effects of CWI such as reduced inflammation (Leeder et al., 2012).

Reduction of intramuscular temperature and its interaction with metabolism has attracted significant research interest. It has been shown that CWI (8°C, 10 min) induces significant decreases in intramuscular temperature (Gregson et al., 2013). Lower intramuscular temperatures are speculated to affect enzymatic activity and rates of intramuscular glycogen synthesis but are also associated with attenuated training adaptions following strength training (Roberts et al., 2015a; Mawhinney and Allan, 2018). However, in cryotherapy research, the most relevant and divisive question pertains to the optimal cooling modality, temperature, and duration to elicit the required physiological response (Costello et al., 2012a). For example, to induce a significant analgesic effect, skin temperature needs to be <13°C for stimulation of neuronal changes, as consistently demonstrated by different research groups (e.g., Bleakley and Hopkins, 2010; Costello et al., 2012a). Currently, one of the most often applied post-exercise CWI protocols comprises a water temperature of around 10°C for a duration of around 10 min (Hohenauer et al., 2015; Vromans et al., 2019). However, the magnitude of heat extraction has been shown to be affected by the subcutaneous adipose tissue thickness, as well as environmental, hormonal, temporal, and nutritional factors and varies between different body parts (Jutte et al., 2001; Costello et al., 2012b; Garami and Székely, 2014; Adams et al., 2015; Romanovsky, 2018; Baker et al., 2020). Despite the increasing number of published articles on CWI and its effectiveness in athletes' recovery, limited studies have focused on the impact of post-exercise CWI on one specific muscle (group) and taking the magnitude of the subcutaneous adipose tissue into account. Previous conducted reviews included various articles with different CWI-protocols (temperature and duration), exercise protocols before CWI, investigated muscles and varying subcutaneous adipose tissue profiles of the participants (Leeder et al., 2012; Hohenauer et al., 2015). The current review article aimed to investigate the effects of one particular post-exercise CWI (i.e., 10 ± 2°C for 10 ± 2 min) on intramuscular temperature changes on one selected muscle (i.e., quadriceps femoris muscle) by taking the skinfold thickness into account.

The collective results may help to improve the estimation of the degree of intramuscular temperature reductions induced by post-exercise CWI on this specific muscle group in various depths. The results of the current study might help health practictioners, coaches, and athletes to estimate the effects of this popular post-exercise CWI protocol on intramuscular temperature changes with respect to the subcutaneous adipose tissue.

## Methods

### Literature Search Strategies and Data Sources

A computerized literature search of online databases was undertaken by one researcher (LF) up to June 2020. The databases searched included PubMed with MeSH Terms (Medical Subject Headings), LIVIVO, Cochrane Library, PEDro, and Google Scholar. The literature search was performed following an *a priori* search strategy using the keywords and combinations presented in Table 1.

**Table 1:** Keywords and Boolean logic combinations.

**Databases**	**Keywords and Boolean logic combinations**
PEDro	cold water immersion and temperature
PubMed, LIVIVO cochrane library	(cold water immersion OR CWI OR cold water immersion therapy OR CWIT OR ice water immersion OR ice baths OR cold water therapy OR cryotherapy OR ice application OR cooling water OR cold treatment) AND [(intramuscle temperature) OR (intramuscular temperature) OR (skeletal temperature) OR (muscle temperature)]
Google scholar	“cold water immersion” AND “intramuscular temperature” AND adipose thickness

### Selection Criteria

Selection criteria were as follows: (1) a defined CWI treatment (10 ± 2°C for 10 ± 2 min) with a minimum immersion depth of the legs (i.e., up to the iliac crest), (2) assessment of intramuscular temperature in the quadriceps femoris muscle before exercise and after the post-exercise CWI intervention at a depth of 1 cm and/or 2 cm and/or 3 cm, (3) exercise protocol of any type before CWI, (4) all participants were healthy, (5) only experimental studies were included, (6) no sex-defined inclusion or exclusion criteria, (7) English and German language restrictions, and (8) the studies measured skinfold thickness of the exercised muscle.

**Figure 1:**
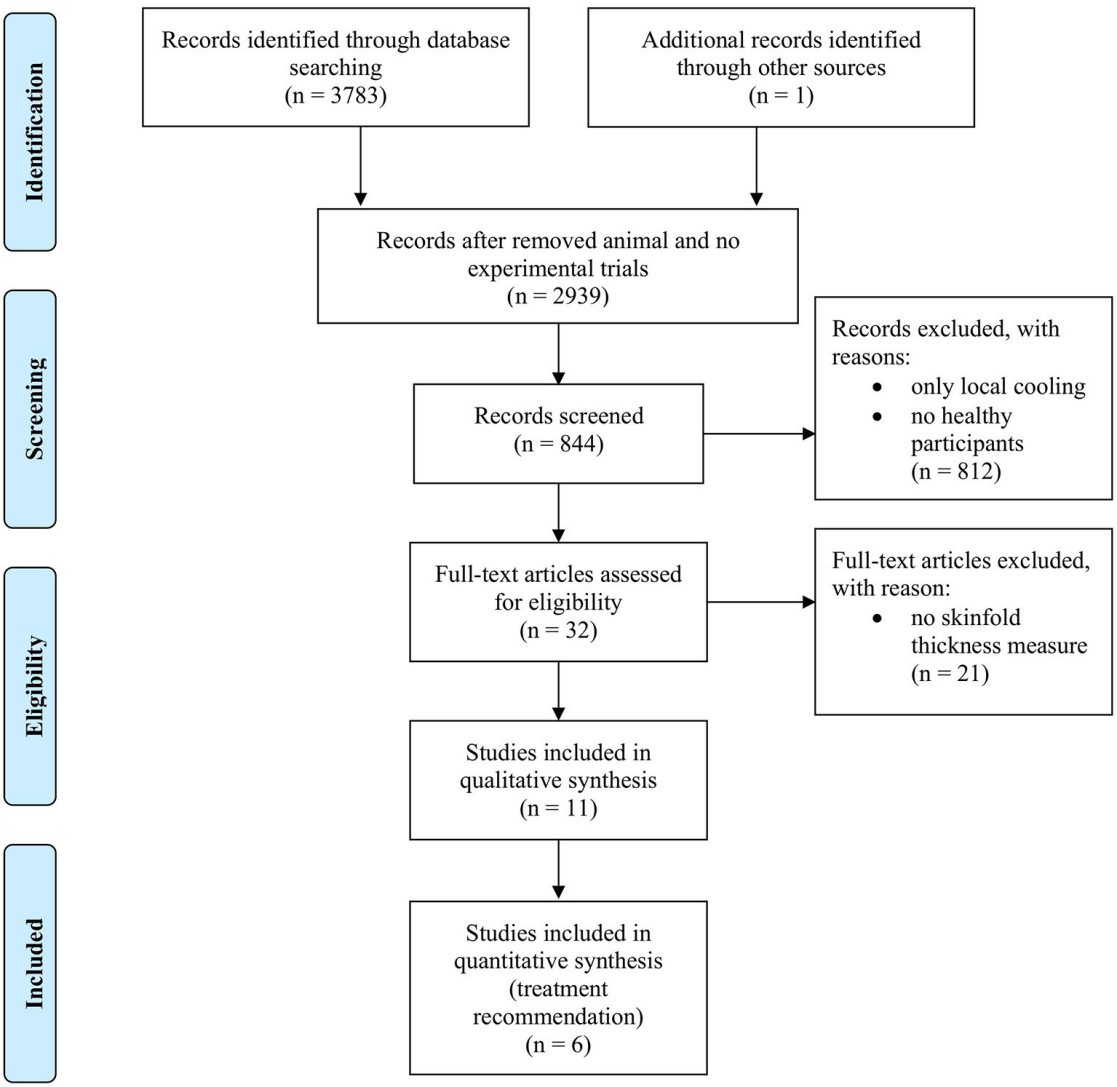
Flowchart describing the systematic search strategy and selection process.

Studies were excluded in case they (1) used cooling techniques other than CWI, (2) combined CWI with any other intervention post-exercise, (3) did not involve human participants, (4) did not use the pre-defined CWI protocol. Figure 1 depicts the systematic search strategy and selection process.

### Data Extraction

General data on the CWI interventions (protocol: °C and min), environmental conditions (°C, % relative humidity), exercise protocol, muscle temperature before and after CWI (including intramuscular temperature at a specific depth), and skinfold thickness (mm) of individual studies were extracted independently by two researchers (LF, EH). In cases where data were missing for the calculations, the authors were contacted by email and, where possible, data were directly extracted from the graphs.

### Risk of Bias

Systematic errors were assessed using the Cochrane's risk of bias tool for both randomized and non-randomized studies (Sterne et al., 2016; Higgins et al., 2019). This strategy has the advantage that risk of bias for different components (domains) is determined separately. Thus, two researchers (LF, EH) independently scored each trial for risk of bias. In case of disagreement, a third researcher (CD) rated the questionable item and agreement was sought by consensus.

Specific domains (random sequence generation, allocation concealment, blinding participants, blinding personnel, blinding outcome assessors, incomplete outcome data, selective reporting, and other bias) were graded for each study. Three options were available for evaluation: low (+) or high (–) risk of bias or “unclear” (?) rating in cases where insufficient information or deficient evidence for bias evaluation was given.

For each non-randomized study, seven domains were rated, including bias due to confounding, selection of participants into the study (pre-intervention), bias in classification of interventions (at intervention) and due to deviations from intended interventions, missing data, measurement of outcomes, and selection of the reported results (post-intervention). Grading options included low risk (+ +), moderate risk (+), serious risk (–), critical risk of bias (− −) and no information (?).

### Data Analysis

Studies were included, if they met all inclusion criteria. Conformity was established in cases where the water temperature and immersion duration did not differ from 10°C and 10 min by more than 2°C and 2 min, respectively, to minimize the impact of protocol variations on intramuscular temperature changes. Means and standard deviations (mean value ± SD) are presented in this study where appropriate. To determine the relationship between subcutaneous adipose tissue and reductions in intramuscular temperature at a specific depth, graphs were plotted with skinfold thickness on the X-axis and changes in intramuscular temperature on the Y-axis. The outermost points were marked and connected to show the extent of the possible effects per depth. The absolute baseline and post-CWI values were applied to estimate mean temperature changes within the quadriceps femoris muscle at a specific muscle depth. Spearman's rho (*r*_s_) was used to evaluate possible correlations between skinfold thickness and intramuscular temperature change with the level of significance set to *p* < 0.05. The analyses were performed in SPSS (Statistical Package for the Social Sciences), version 26.0 (SPSS Inc, Chicago, IL, USA). Because control groups were very heterogeneous (e.g., “active recovery,” “passive rest,” “whole body cryotherapy”) it was decided to meta-analyse the pre-post intamuscular temperature data of the intervention arms of the different studies only. This decision enabled the inclusion of the single-group Rech (2013) study. Meta-analyses with random-effects model were used to examine the overall weighted mean effect size of post-exercise CWI on intramuscular temperature for each measurement depth (1, 2, 3 cm). Inverse-variance method was used to calculate the weighting factors. Meta-Analyses of the pre-post intramuscular temperature data were calculated assuming a correlation coefficient of 0.7 (Borenstein et al., 2009). To assess the robustness of the overall weighted estimate, sensitivity analyses with correlation coefficients of 0.5 and 0.9, respectively, were conducted (Borenstein et al., 2009). The Cochran Q statistic and its corresponding *p*-value, as well as *I*^2^ were calculated to assess across studies' heterogeneity and its degree, respectively. Higgins suggested benchmarking values for the interpretation of *I*^2^ as followed: *I*^2^ around 25% (low heterogeneity), *I*^2^ around 50% (moderate heterogeneity) and *I*^2^ around 75%, or more (high heterogeneity) (Higgins and Thompson, 2002). The Comprehensive Meta-Analysis 2 software (CMA- Version 2 Professional, Biostat Inc., Englewood, USA) was used for the calculations of the weighted overall effect size, the corresponding 95% confidence intervals (95% CI), the sensitivity analyses and to establish the forest plots.

## Results

### Risk of Bias rating

**Figure 2:**
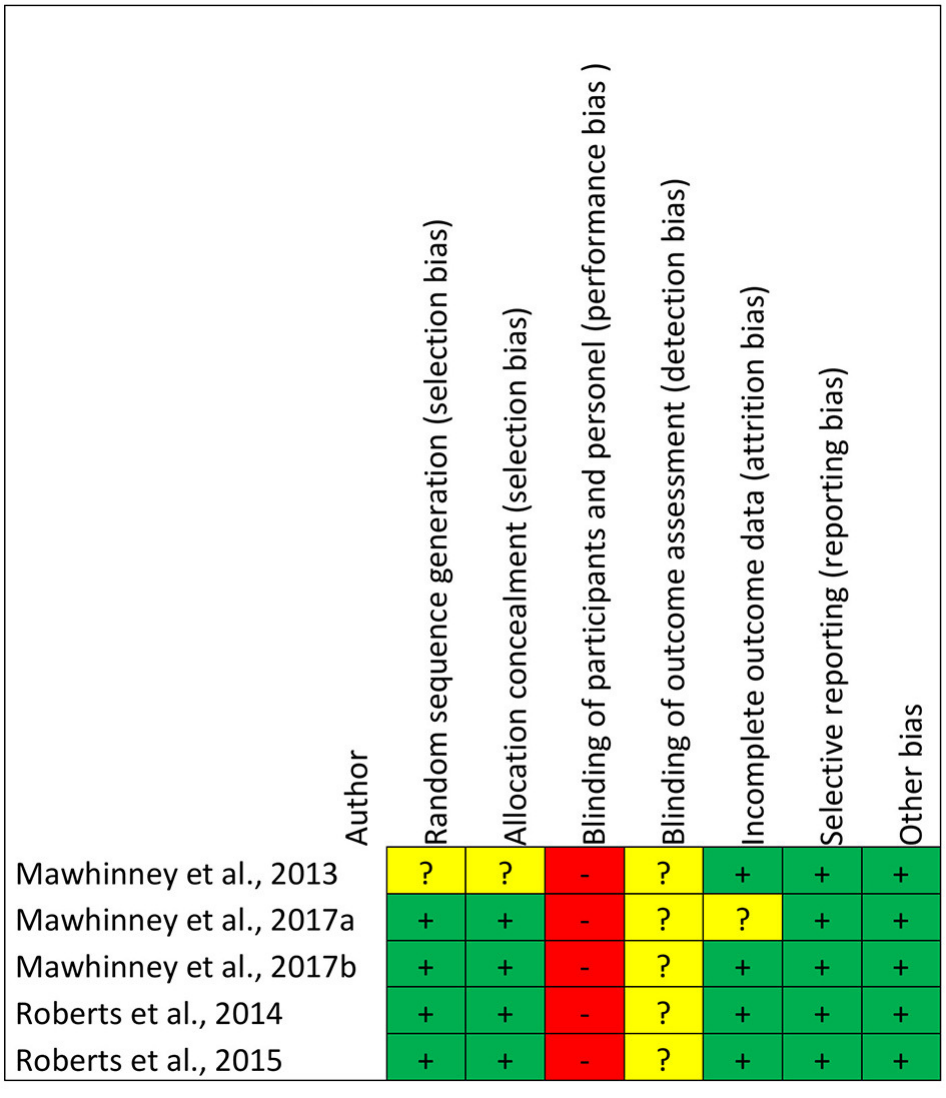
Risk of bias graph for the *n* = 5 included randomized studies (*n* = 1 non-randomized study not included).

**Figure 3:**
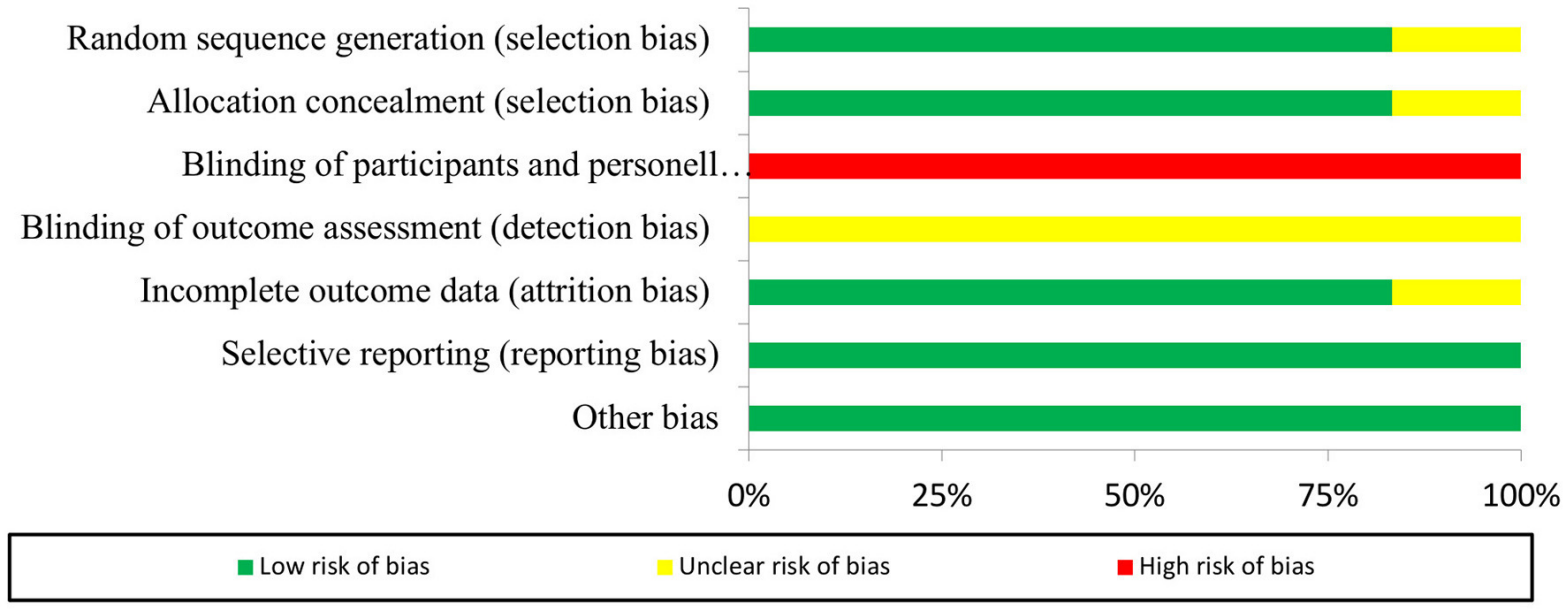
Risk of bias summary for the *n* = 5 included randomized studies (*n* = 1 non-randomized study not included).

The Cochrane's risk of bias tool for randomized studies was used in five studies (Mawhinney et al., 2013, 2017a,b; Roberts et al., 2014, 2015b). Risk of bias analysis demonstrated high risk of performance bias and unclear risk of detection bias. Low risk of selection, attrition, reporting, and other bias was obtained for all five studies. Comprehensive details of risk of bias for collective and individual studies are presented in Figures 2, 3.

One study was rated with the ROBINS-I tool for assessing risk of bias in non-randomized studies of interventions (Rech, 2013). In this case, low risk of bias was observed for selection of results and missing data. Moderate risk of bias was reported for confounding selection of participants, classification of intervention, and measurement of outcomes, and serious risk of bias for deviation from intended interventions.

### Included Studies

A total of *n* = 11 articles were taken into consideration for the analysis (Gregson et al., 2013; Mawhinney et al., 2013, 2017a,b; Rech, 2013; Broatch et al., 2014; Roberts et al., 2014, 2015b; Solianik et al., 2015; Joo et al., 2016; Choo et al., 2018). However, after screening for the quantitative analysis, only *n* = 6 studies met all of our inclusion criteria and were used for further evaluation (Mawhinney et al., 2013, 2017a,b; Rech, 2013; Roberts et al., 2014, 2015b). The total study population included *n* = 70 healthy volunteers. The majority of selected studies was performed on recreationally or physically active male volunteers. In one study, including *n* = 16 volunteers, sex distribution and fitness status was not specified (Rech, 2013). The mean sample size was *n* = 11.66 (range: 10–16 volunteers) and mean age (± SD) of the total study population was 23.5 ± 3.25 years. The characteristics of the included studies are summarized in Table 2.

**Table 2:** Summary of included studies.

**References**	**Sample size (*n*), sex, and** **age**	**EC**	**CWI protocol: water temperature (^**°**^C) and duration (min)**	**Exercise protocol**	**Intramuscular measurement**	**SFT in mm (mean ± SD)**	**Intramuscular temperature in ^**°**^C baseline (mean ± SD)**	**Intramuscular temperature in ^**°**^C post-treatment (mean ± SD)**
Mawhinney et al. (2013)	*n =* 12 male 25.5 ± 4.7 yr	22–24°C	8°C for 10 min (up to the iliac crest)	Cycling at 70% VO_2max_ until Tcore reaches 38°C	MQF VL, 3.0 cm (plus 1/2 of SF), 2.0 and 1.0 cm depth	11.42 ± 2.65	3.0 cm = 36.27 ± 0.37 2.0 cm = 35.85 ± 0.43 1.0 cm = 34.98 ± 0.66	3.0 cm = 36.58 ± 0.64 2.0 cm = 34.49 ± 1.09 1.0 cm = 31.10 ± 1.68
Mawhinney et al. (2017a)	*n =* 12 male 26 ± 6 yr	22–24°C	8°C for 10 min. (up to the iliac crest)	4 × 10 maximum squat exercise with 2 min. rest	MQF VL, 3.0 cm (plus 1/2 of SF), 2.0 and 1.0 cm depth	10.79 ± 2.73	3.0 cm = 36.19 ± 0.32, 2.0 cm = 35.69 ± 0.45, 1.0 cm = 34.92 ± 0.59	3.0 cm = 34.58 ± 0.9 2.0 cm = 32.04 ± 1.38 1.0 cm= 29.48 ± 1.47
Mawhinney et al. (2017b)	*n =* 10 male 22.3 ± 3.4 yr	22–24°C ~40% rh	8°C for 10 min. (up to iliac crest)	Cycling at 70% VO_2max_ until Tcore reaches 38°C	MQF VL, 3.0 cm (plus 1/2 of SF), 2.0 and 1.0 cm depth	15.65 ± 7.58	3.0 cm = 36.14 ± 0.66 2.0 cm = 35.53 ± 0.81 1.0 cm = 34.80 ± 0.90	3.0 cm = 35.57 ± 0.49 2.0 cm = 33.09 ± 1.36 1.0 cm = 30.94 ± 1.51
Rech (2013)	*n =* 16 subjects 24.3 ± 1.84 yr	unknown	10°C (up to iliac crest) until Tm decreased 7°C below pre-exercise (= cooling rate 0.27 ± 0.18°C/min) or 30 min	Cycling for 30 min. (HR130-150 bts/min.)	MQF rectus femoris, 2.0 cm sub-adipose	11.0 ± 3.7	35.3 ± 1.2	30.8 ± 3.7
Roberts et al. (2014)	*n =* 10 male 21.3 ± 1.6 yr	24.3 ± 0.6°C 48.6 ± 1.2% rh	10.0 ± 0.3°C for 10 min. (up to the clavicle)	High intensity resistance training for 1 h: 6 sets of 8, 8, 10, 12, 10, and 10 front and back squats, 3 × 12 walking dumbbell lunges, 3 × 12 countermovement drop jumps	MQF VL, 1.0 cm depth	6.4 ± 3.1	35.2 ± 0.5	32.99 ± 4.64
Roberts et al. (2015a)	*n =* 10 male 21.4 ± 2.0 yr	24.4 ± 0.2°C 43.5 ± 1.6% rh	10.0 ± 0.2°C for 10 min. (up to umbilicus)	Maximal unilateral isokinetic knee extensor exercise 10 × 20 reps with 2 min. rest	MQF VL, 1.15 cm depth	6.5 ± 3.4	34.5 ± 0.9	28.1 ± 6.1

*EC, Environmental conditionsl; HR, heart rate; MQF, M. quadriceps femoris; rh, relative humidity; SF, skinfold; SFT, skinfold thickness; Tcore, core temperature; Tm, Temperature; VL, vastus lateralis; yr, year*.

The remaining five studies were excluded from the quantitative analysis as the CWI protocol did not meet conformity (water temperature at 10 ± 2°C and treatment duration 10 ± 2 min) or data reporting was incomplete. Three studies used CWI protocols ranging between 8 and 14°C for 5 min (Choo et al., 2018) and 10°C for 15 min (Broatch et al., 2014) or 14°C for 20 min (Solianik et al., 2015). Furthermore, we were unable to extract all necessary data from two studies that employed a CWI protocol using cold water at 8°C for the duration of 10 min and 2 × 5 min (Gregson et al., 2013; Joo et al., 2016).

The reasons for exclusion were, that variations of the CWI protocols (temperature and duration) would have led to potential under- or overestimations of the impact of the CWI protocol on muscle cooling, whilst incomplete data reporting of the skinfold thickness would have made it not possible to evaluate the influence of the subcutaneous adipose thickness on the intramuscular cooling rate.

#### Environmental Conditions

Environmental temperature and relative humidity were between 22 and 24.4°C (mean 23.54°C) and between 40 and 48.6% (mean 44.0%) in the included studies. One study did not provide information on the environmental conditions (Rech, 2013).

#### Exercise Program

Participants had to perform a submaximal cycling endurance protocol in three studies (Mawhinney et al., 2013, 2017b; Rech, 2013) and maximum or high-intensity exercises (Roberts et al., 2014, 2015b; Mawhinney et al., 2017a) prior to the CWI intervention. Two studies used a cycling protocol at 70% VO_2max_ until the core temperature reached 38°C (Mawhinney et al., 2013, 2017b). In one study, participants were required to cycle for 30 min and heart rates between 130 and 150 bpm were recorded (Rech, 2013). Maximum or high-intensity exercises were carried out as follows: 4 × 10 maximal squat exercises with a 2 min break between sets (Mawhinney et al., 2017a), 10 × 20 maximal isokinetic knee extension exercises with a 2 min rest (Roberts et al., 2015b) and high-intensity resistance training lasting for around 1 h (Roberts et al., 2014).

#### Skinfold Thickness

The mean skinfold thickness for all six studies was 10.29 ± 3.86 mm, ranging between 6.40 and 15.65 mm. One study used an ultrasound device to determine the subcutaneous adipose tissue above the assessed muscle (Rech, 2013). The remaining five studies used a Harpenden skinfold caliper and divided the result by two to determine the subcutaneous adipose tissue thickness (Mawhinney et al., 2013, 2017a,b; Roberts et al., 2014, 2015b). Skinfold thickness of the thigh was measured 5 cm proximal from the patella (Roberts et al., 2015b), 15 cm proximal from the superior margin of the patella (Mawhinney et al., 2013, 2017a), mid-way between the inguinal crease and the patella (Rech, 2013; Roberts et al., 2014) and was not described in one study (Mawhinney et al., 2017a).

#### Intramuscular Temperature Measurement

Five studies investigated the intramuscular temperature of the vastus lateralis of the quadriceps femoris muscle (Mawhinney et al., 2013, 2017a,b; Roberts et al., 2014, 2015b) while one evaluated the temperature in the rectus femoris (Rech, 2013).

Intramuscular temperature was assessed using an implantable probe (Roberts et al., 2015b), fine wire thermistor or needle thermistor (Mawhinney et al., 2013, 2017a,b; Roberts et al., 2014) or a thermocouple probe (Rech, 2013). Two studies assessed intramuscular temperature at a frequency of 1 Hz (Roberts et al., 2014, 2015b). In one investigation, data acquisition was conducted at ~0.03339 Hz (Rech, 2013) while the exact measurement frequency was unclear in the remaining three studies (Mawhinney et al., 2013, 2017a,b).

### Differences in Intramuscular Temperature

**Figure 4:**
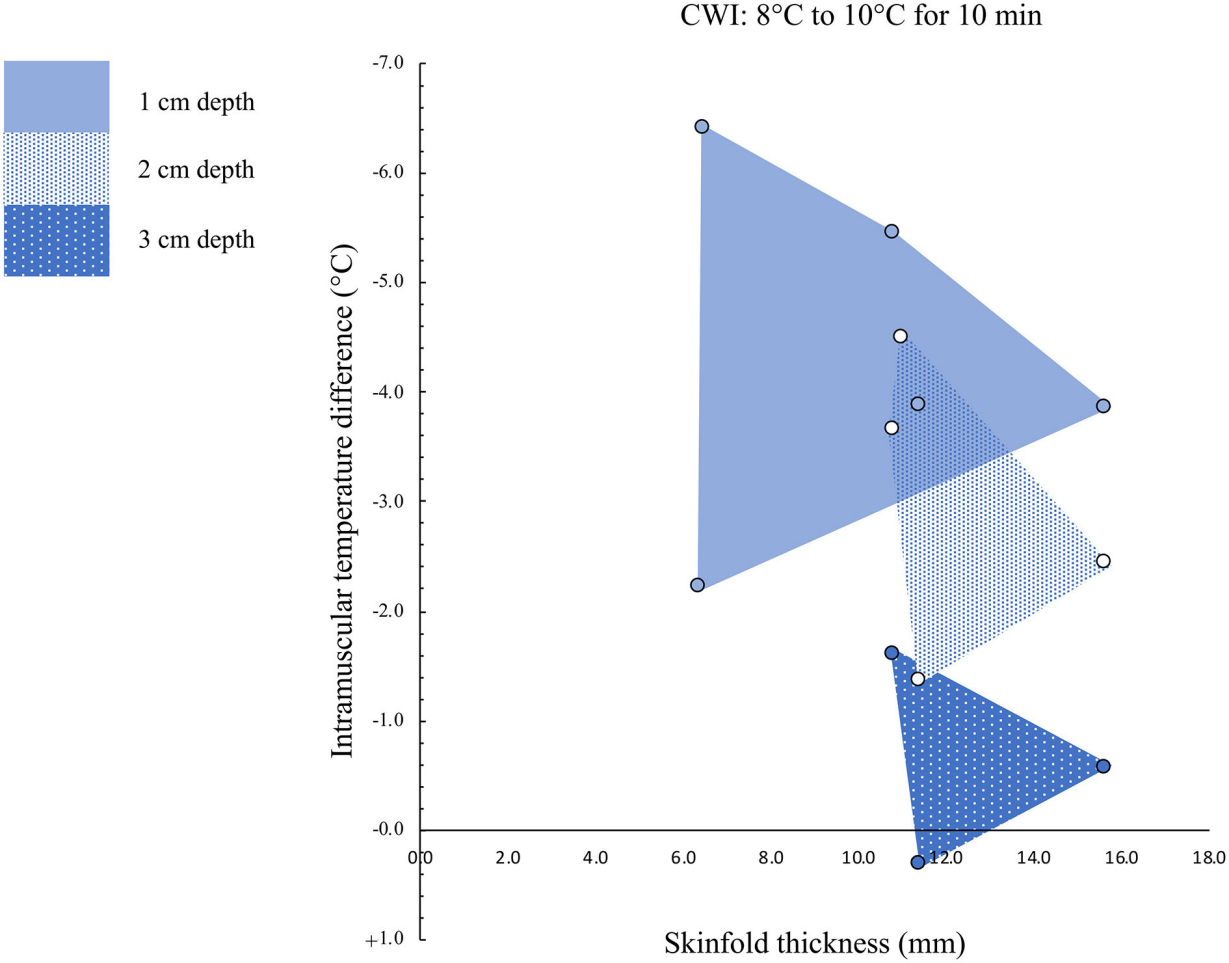
Intramuscular temperature changes in relation to the skinfold thickness. Muscle temperature was measured at depths of 1, 2, and 3 cm.

The intramuscular temperature changes are presented in Figure 4.

#### Intramuscular Temperature Differences at a Muscle Depth of 1 cm

**Figure 5:**
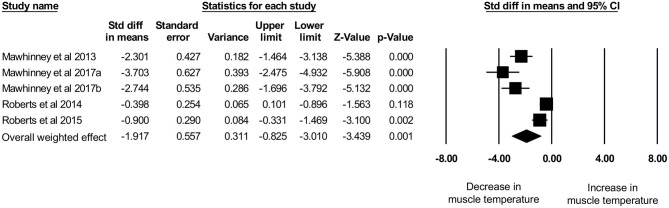
Forest plot of the meta-analysis illustrating the comparison between baseline and post-exercise CWI temperature values at a muscle depth of 1 cm.

Five studies, with a total sample size of *n* = 54 participants, investigated the effects of CWI on intramuscular temperature at a depth of 1 cm in the vastus lateralis of the quadriceps femoris muscle (Mawhinney et al., 2013, 2017a,b; Roberts et al., 2014, 2015b). The mean water temperature was 8.8 ± 1.0°C for a mean duration of 10 ± 0 min. We observed an intramuscular temperature decrease of 4.36 ± 1.61°C (range: −2.21°C to −6.40°C) at a mean skinfold thickness of 10.15 ± 3.86 mm (range: 6.40–15.65 mm). No correlation (*p* = 1.0) between skinfold thickness and intramuscular temperature reduction was observed (*r*_s_ = 0.0). The use of CWI decreased intramuscular temperature significantly (Figure 5) between baseline and post-exercise CWI measurements (standardized differences in means (*d*) = −1.92 [95% CI: −3.01 to −0.83]), based on this limited set of published studies. High and statistically significant heterogeneity was observed (Q = 41.86, df (*Q*): 4, *p* = 0.001; I^2^: 90.4%). After conducting sensitivity analyses using correlation coefficients of 0.5 and 0.9, the results remained statistically significant and in favor of for reduced intramuscular temperature after CWI *d* = −2.12 [95% CI: −3.32 to −0.92] and *d* = −1.37 [95% CI: −2.20 to −0.55], respectively.

#### Intramuscular Temperature Differences at a Muscle Depth of 2 cm

**Figure 6:**
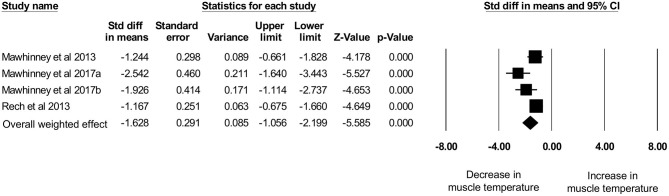
Forest plot of the meta-analysis illustrating the comparison between baseline and post-exercise CWI temperature values at a muscle depth of 2 cm.

Four studies, with a total sample size of *n* = 50 participants, evaluated the effects of CWI on intramuscular temperature at a depth of 2 cm in the vastus lateralis of the quadriceps femoris muscle (Mawhinney et al., 2013, 2017a,b) and rectus femoris (Rech, 2013). The mean water temperature was 8.5 ± 1.0°C for a mean duration of 10.0 ± 0.0 min. The intramuscular temperature decrease was 2.98 ± 1.37°C (range: −1.36 to −4.50°C) at a mean skinfold thickness of 12.21 ± 2.30 mm (range: 10.79–15.65 mm). No correlation (*p* = 0.2) between skinfold thickness and intramuscular temperature reduction was observed (*r*_s_ = −0.8). Based on this limited set of published studies, post-exercise CWI reduced intramuscular temperature significantly, which can be seen in Figure 6 (*d* = −1.63 [95% CI: −2.20 to −1.06]). Moderate and statistical significant heterogeneity was observed [*Q* = 8.66, df (*Q*): 3, *p* = 0.034, *I*^2^: 65.3%]. After the sensitivity analysis with a correlation coefficient of 0.5 and 0.9 results remainded statistically significant in favor for decreased muscle temperature after CWI with *d* = −1.79 [95% CI: −2.41 to −1.17] and *d* = −1.16 [95% CI: −1.60 to −0.73], respectively.

#### Intramuscular Temperature Differences at a Muscle Depth of 3 cm

**Figure 7:**
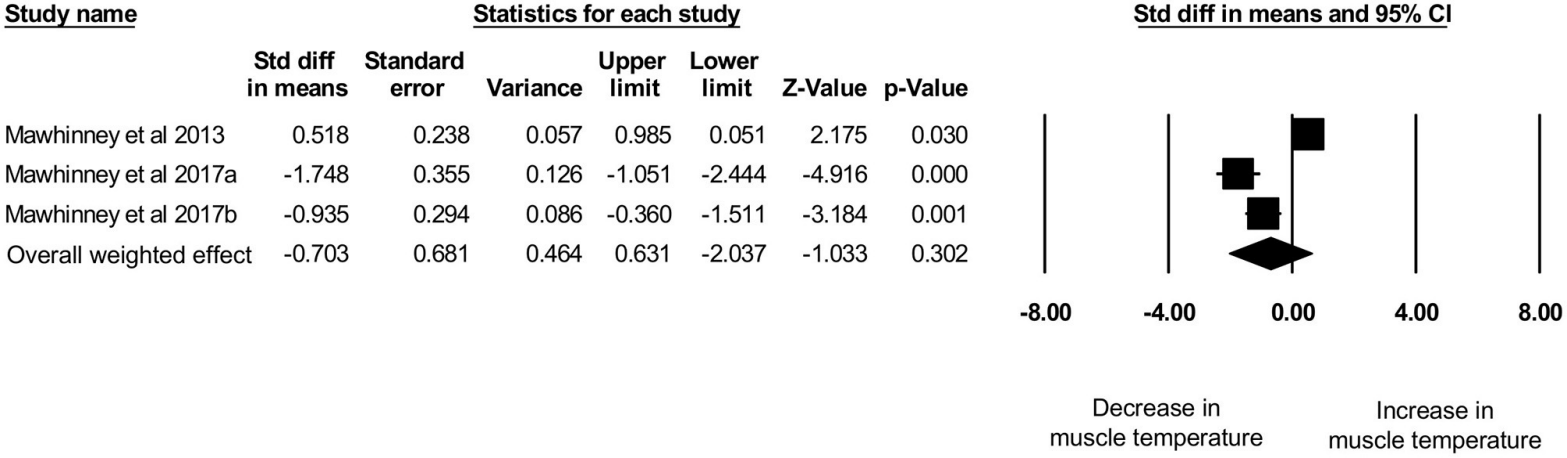
Forest plot of the meta-analysis illustrating the comparison between baseline and post-exercise CWI temperature values at a muscle depth of 3 cm.

Three studies, with a total sample size of *n* = 34 participants, investigated the effects of CWI on intramuscular temperature at a depth of 3 cm in the vastus lateralis of the quadriceps femoris muscle (Mawhinney et al., 2013, 2017a,b). The mean water temperature was 8.0 ± 0.0°C for a mean duration of 10.0 ± 0.0 min. The mean intramuscular temperature decrease was 0.62 ± 0.96°C (range: −1.61 to + 0.31°C) at a skinfold thickness of 12.62 ± 2.64 mm (range: 10.79–15.65 mm). No correlation (*p* = 0.6) between skinfold thickness and intramuscular temperature reduction was observed (*r*_s_ = −0.5). Based on this limited set of published studies, post-exercise CWI reduced intramuscular temperature albeit not statistically significant (Figure 7) with *d* = −0.70 [95% CI: −2.04 to 0.63]. High and statistically significant heterogeneity was observed (*Q* = 32.54, df (*Q*): 2, *p* < 0.001, *I*^2^: 93.9%). After the sensitivity analysis with a correlation coefficient of 0.5 and 0.9 results remainded in favor for decreased muscle temperature after CWI albeit statistically not significant with *d* = −0.77 [95% CI: −2.25 to 0.7] and *d* = −0.52 [95% CI: −1.50 to 0.46], respectively.

## Discussion

The main aim of this systematic review is to provide an overview on intramuscular temperature changes at different depths in the quadriceps femoris muscle for a well-established post-exercise CWI protocol (10°C for 10 min), taking subcutaneous adipose tissue into account. Our results demonstrate that CWI reduces intramuscular temperature after exercise significantly in the upper muscle layers by around 4°C at 1 cm (*p* = 0.001) and by 3°C at 2 cm (*p* < 0.001) but not at a depth of 3 cm (1°C, *p* = 0.304). Reduction in muscle tissue temperature was not significantly correlated to subcutaneous thickness measurements. Furthermore, potential interindividual changes in muscle tissue temperature at all measurement depths (i.e., 1, 2, and 3 cm) can be seen in Figure 4 and are also reflected in the Figures 5–7. In general, we saw most pronounced temperature drops after CWI at superficial muscle depth (1 cm) that were progressively alleviated in deeper layers (2 and 3 cm). At 3 cm, muscle temperature was often close to baseline values. In one study, the temperature was even above the baseline value after exercises (Mawhinney et al., 2013). This result is not surprising taking into account that heat loss differs between superficial and deep tissues due to different temperature gradients. Skin temperature can be easily and rapidly decreased due to direct contact between the tissue and the cooling modality. The relatively large temperature differences between skin tissue and cooling modality can lead to a considerable drop in skin temperature. In muscle tissue, heat loss takes place indirectly via conduction to overlying cooler tissues. The temperature gradient allows transfer of heat from deep to superficial tissues, but the magnitude of heat loss is obviously smaller in tissues at greater depth (Merrick et al., 2003). Consequently, with greater heat produced during exercise in deep muscle tissues, more cooling time is required to decrease the intramuscular temperature below baseline values. Indeed, the cooling time might have been too short in the study of Mawhinney et al. (2013), where the intramuscular temperature was even after the CWI treatment 0.31°C higher compared to baseline at a depth of 3 cm. In the remaining *n* = 2 studies, the cooling time was sufficient to decrease the muscle tissue temperature at a depth of 3 cm by 1.61 and 0.56°C (Mawhinney et al., 2017a,b). However, it has to be mentioned that the post-CWI data showing only a 0.57°C reduction was collected 10 min after the cold treatment (Mawhinney et al., 2017b). Although the afterdrop has to be taken into account, it is possible that maximal intramuscular temperature reduction had already occurred at an earlier time-point in this study, which would result in underestimation of the magnitude of muscle temperature decrease in this case.

The subcutaneous fat layer, which varied in our included studies from 6.40 mm (Roberts et al., 2014) to 15.65 mm (Mawhinney et al., 2017b) has been assumed to play a large role to limit muscle temperature reductions (Jutte et al., 2001; Otte et al., 2002). Although skinfold thickness of the included recreationally active males in the studies was measured, different body composition (ecto-, meso und endomorphy) might be an additional confounding factor (Stephens et al., 2018). Compared to muscle tissue, adipose tissue has low thermal conductivity (0.23 vs. 0.46 k) and thus, serves as a physiological heat insulator (El-Brawany et al., 2009). From this perspective, an inverse relationship between subcutaneous adipose tissue thickness and intramuscular temperature decrease was expected. However, our results suggest that lower skinfold thickness values do not guarantee higher reductions in intramuscular temperature after cooling, indicating that intraindividual factors such as microvascular blood flow or intramuscular perfusion must be additionally considered (Mawhinney et al., 2013). Indeed, it has been shown that variations in skin microvasculature and muscle perfusion are known to be normally present (Mayrovitz et al., 1997; Mawhinney et al., 2020). Additionally, it has been demonstrated, that in cold adapted humans, cold exposure is subjectively considered less stressful and that the physiological responses to cold are attenuated (Rintamaki, 2007). Consequently, the included studies in the current review were carried out in different countries and the environmental conditions (season when the experiments were carried out) might have had a significant impact on the physiological responses to the CWI. Also the type of exercise and muscle contraction, potentially influence the magnitude of heat extraction from muscle tissue. In our review, three of the six included studies focused on performance of a high-intensity exercise task (Roberts et al., 2014, 2015b; Mawhinney et al., 2017a) and submaximal cycle ergometer tests prior to CWI (Mawhinney et al., 2013, 2017a; Rech, 2013). Different mechanical load/resistance programs might have caused active muscle to produce more or less energy in the form of work and heat, derived from chemical reactions (Yamada, 2017). Further research and analysis with individual customized exercise protocols is necessary to evaluate the potential relationship between the type of exercise task and intramuscular temperature development.

Our results revealed that a CWI protocol (10 ± 2°C, 10 ± 2 min) maximally lowered the intramuscular temperature of the quadriceps femoris muscle to 6.40°C at a depth of around 1 cm, 3.65°C at around 2 cm, and 1.61°C at around 3 cm. From a rehabilitation perspective, the impacts of even these maximum temperature reductions may be too small to decrease cellular metabolism to a clinically significant extent for protection of damaged muscle tissue from secondary ischemic and enzymatic injuries (Merrick, 2002; Bleakley and Hopkins, 2010). Research on animal models has demonstrated that cellular metabolism is optimally reduced when tissue temperatures between 5 and 15°C are reached (Osterman et al., 1984; Merrick, 2002). In our review, the minimum intramuscular temperatures post-exercise CWI were around 28°C at a depth of 1 cm, 30°C at 2 cm and 32°C at 3 cm (Rech, 2013; Roberts et al., 2015b; Mawhinney et al., 2017a). Although the ability of water to extract heat from the body is extremely high (24 times higher than air), other materials should be considered when the primary aim is heat extraction from muscle tissue. For example, ice possesses a four times higher heat transfer coefficient than water, which is related to phase changes during the melting process (Bleakley et al., 2014). However, the potential positive effects of cold water on cellular metabolism reductions and further of enhanced muscle recovery after exercise (e.g., DOMS) may not be primarily attributed to its intramuscular temperature reduction effect (Wilcock et al., 2006). Indeed, the impact of the cold water on muscle strength was shown to be no greater than thermoneutral placebo water immersion (34.7°C) after a high-intensity interval training session (Broatch et al., 2014). Hydrostatic pressure leading to intracellular-intravascular fluid shifts, reduction of edema and increased cardiac output may also play an important role in enhancing muscle recovery. Immersion in cool and thermoneutral water could provide comparable recovery results unless soft tissue injuries have occurred, in which case cooled tissue may provide greater benefits (Sellwood et al., 2007; Mutlu and Yilmaz, 2020).

Another variable that may have a positive effect on post-exercise recovery is immersion depth of the body, although this variable was not investigated in the current study. The hydrostatic pressure, which varies with immersion depth, causes displacement of body fluids from the extremities to the central cavity. As a result of exercise or muscle damage, oxygen delivery by these fluids is reduced to localized cells, leading to increased cellular damage or death (Friden and Lieber, 2001). Post-exercise water immersion of the body (not exclusively cold water) may reduce the occurrence of potential cell-damaging edema and inflammation by increasing the pressure gradient between the interstitial and intravascular space and promoting re-absorption of interstitial fluid, similar to compression stockings (Partsch et al., 2004). A combination of cold water and high hydrostatic pressure (e.g., through immersing the body up to the clavicle) could act synergistically, as decreased muscle temperature may reduce edema formation through suppression of muscle perfusion and fluid diffusion into the interstitial space (Yanagisawa and Fukubayashi, 2010; Mawhinney et al., 2017b; Tipton et al., 2017). Lower intramuscular temperatures can also lead to reduced inflammatory markers like creatine-kinase after exercise induced muscle damage and therefore might attribute to muscle function recovery (Eston and Peters, 1999). Additionally, after exercise induced muscle damage, maximum voluntary isometric contraction has been shown to recover faster after CWI compared to a control group (Machado et al., 2017). However, as seen in our results, it is questionable if significant temperature reductions can be achieved in deep muscle tissue with this protocol and as a result, if inflammatory responses can be reduced in these deep tissue layers. However, we have to consider, that also non-significant differences might make the difference between winning and loosing in a high athletic population.

Although one of the most popular post-exercise CWI protocol was used for evaluation in this study, only a small number of studies could be identified for the current analysis. Taking this into consideration, the analyses and results which are based on a limited set of published studies, should be interpreted with caution as publication bias can't be excluded. The results are also limited to this investigated CWI protocol and can't be transferred to other (post-exercise) CWI protocols. However, this also highlights the need for further (post-exercise) CWI studies in this field with specific inclusion criteria to further assess the impact of this recovery strategy. Further studies using standardized exercise protocols, identifying further key variables and taking the above mentioned key variables into account are warranted, to evaluate the tissue cooling magnitude of specific CWI protocols on different muscle groups in a well-defined population.

## Conclusion

In conclusion, the collective findings of this review indicate that post-exercise CWI (10 ± 2°C for 10 ± 2 min) decreases intramuscular temperature in the quadriceps femoris muscle significantly in the upper muscle layers (1, 2 cm). However, the intramuscular temperature reductions in the quadriceps femoris muscle showed a wide variation and the subcutaneous adipose tissue did not significantly correlate to the temperature reduction. Beside skinfold thickness, additional key variables like the intensity and length of the exercise protocols, intramuscular perfusion and the investigated population itself might have a significant influence on the magnitude of intramuscular heat extraction during post-exercise CWI.

## Data Availability Statement

The original contributions presented in the study are included in the article/Supplementary Material, further inquiries can be directed to the corresponding author/s.

## Author Contributions

EH, LF, and RC: conceived and designed the study. LF, EH, JT, RC, and CD: analyzed the data. CD, EH, LF, RC, WT, and JT: wrote the paper. All authors: contributed to the article and approved the submitted version.

## Conflict of Interest

The authors declare that the research was conducted in the absence of any commercial or financial relationships that could be construed as a potential conflict of interest.
